# A Thalamic-Fronto-Parietal Structural Covariance Network Emerging in the Course of Recovery from Hand Paresis after Ischemic Stroke

**DOI:** 10.3389/fneur.2015.00211

**Published:** 2015-10-13

**Authors:** Eugenio Abela, John H. Missimer, Andrea Federspiel, Andrea Seiler, Christian Walter Hess, Matthias Sturzenegger, Roland Wiest, Bruno J. Weder

**Affiliations:** ^1^Support Center for Advanced Neuroimaging (SCAN), Institute for Diagnostic and Interventional Neuroradiology, University Hospital Inselspital, University of Bern, Bern, Switzerland; ^2^Laboratory of Biomolecular Research, Paul Scherrer Institute, Villigen, Switzerland; ^3^Department of Psychiatric Neurophysiology, University Hospital of Psychiatry, University of Bern, Bern, Switzerland; ^4^Department of Neurology, University Hospital Inselspital, University of Bern, Bern, Switzerland; ^5^Department of Neurology, Kantonsspital St. Gallen, St. Gallen, Switzerland

**Keywords:** stroke recovery, structural covariance network, fronto-parietal network, thalamocortical loop, tensor-based morphometry

## Abstract

**Aim:**

To describe structural covariance networks of gray matter volume (GMV) change in 28 patients with first-ever stroke to the primary sensorimotor cortices, and to investigate their relationship to hand function recovery and local GMV change.

**Methods:**

Tensor-based morphometry maps derived from high-resolution structural images were subject to principal component analyses to identify the networks. We calculated correlations between network expression and local GMV change, sensorimotor hand function and lesion volume. To verify which of the structural covariance networks of GMV change have a significant relationship to hand function, we performed an additional multivariate regression approach.

**Results:**

Expression of the second network, explaining 9.1% of variance, correlated with GMV increase in the medio-dorsal (md) thalamus and hand motor skill. Patients with positive expression coefficients were distinguished by significantly higher GMV increase of this structure during stroke recovery. Significant nodes of this network were located in md thalamus, dorsolateral prefrontal cortex, and higher order sensorimotor cortices. Parameter of hand function had a unique relationship to the network and depended on an interaction between network expression and lesion volume. Inversely, network expression is limited in patients with large lesion volumes.

**Conclusion:**

Chronic phase of sensorimotor cortical stroke has been characterized by a large scale co-varying structural network in the ipsilesional hemisphere associated specifically with sensorimotor hand skill. Its expression is related to GMV increase of md thalamus, one constituent of the network, and correlated with the cortico-striato-thalamic loop involved in control of motor execution and higher order sensorimotor cortices. A close relation between expression of this network with degree of recovery might indicate reduced compensatory resources in the impaired subgroup.

## Introduction

As both cross-sectional and a few longitudinal observational studies have demonstrated, behavioral recovery from hemiparesis after ischemic stroke shows marked between-subject variability (1, 2). This variability is thought to be determined not only by general demographic or clinical factors – such as age, gender or medical comorbidities – but also by neurobiological processes prompted by damage to critical nodes of functional and structural brain networks (3, 4). Activation studies using functional MRI (fMRI) have contributed considerably in the past to current knowledge of these processes (5–7); moreover, resting state fMRI and structural MRI have provided complementary insights in recent years (8). The improved understanding of stroke provided by neuroimaging could impact neurorehabilitative therapies (9–11).

Activation studies performed with fMRI have shown that successfully recovered subjects show almost normal cerebral patterns, exhibiting change during recovery from attention demanding controlled processing of motor performance in the subacute stage to more fluent and automatic processing in the late chronic stage (12). This suggests recovery at the synaptic and/or neuronal level in the perilesional zone. In contrast, individuals presenting impaired recovery retain ineffective motor patterns and may not regain fully the specific motor function (13, 14); they, thus, possibly require cognitive control and concentrated effort to maintain motor execution (15). Accordingly, volitional and emotional effort are means to enhance output in a diseased, low-efficient motor system, as indicated by the enhanced activation of motor networks observed in fMRI-studies of patients with chronic motor impairment (12). An additional aspect of the recovery process evidenced by studies at varying stages post-stroke is the influence of the contralesional hemisphere, functionally rather supporting motor activity in the early acute phase and mainly inhibiting it in the chronic stage (16, 17).

In the following, we utilize structural MRI to study stroke recovery in a patient cohort of 28 patients selected for first cortical sensorimotor stroke and associated initial hand paresis or plegia. The analysis employs a relatively new method, tensor-based morphometry (TBM), to quantify gray matter volume (GMV) changes during recovery (18, 19). While indicating structural neuronal plasticity, the changes cannot be assigned *in vivo* to a specific mechanism, e.g., axon sprouting, dendritic branching or synaptogenesis (20). Requiring high-resolution MRI [3D modified driven equilibrium Fourier transform (3D-MDEFT)] imaging, TBM evaluates the transformations relating one acquisition to a second in a single subject. In our longitudinal study, the first acquisition was performed after 3 months in the subacute phase and the second after 9 months in the chronic phase. In all patients, initial diffusion-weighted MR images (21) delineated impacted critical brain lesions. High-resolution T1 (3D-MDEFT)-MRIs were acquired in 28 patients 3 and 9 months after stroke (22). An example of multimodal imaging in a wider sense (23), the acquisition protocols provided two non-redundant data sets from the same MR instrument in the same study population: bright tissue contrast for lesion delineation in the acute phase and GMV changes derived from the high-resolution T1 images by TBM analysis.

Accompanying the imaging was an array of clinical, motor and sensory assessments performed regularly during the 9-month study. Of the behavioral assessments, picking small objects (PSO), a lateralized motor skill requiring a particular precision grip, showed the greatest variance over the 9-month trial period (21). Response feature analysis (RFA) using Akaike’s information criterion applied to the 9-month recovery trajectories of the individual patient tests partitioned the patient cohort into three subgroups showing fast linear, slow exponential or impaired recovery (24). A multivariate analysis, principal component analysis (PCA), of the PSO task confirmed the partitioning among the 28 patients and characterized each patient’s expression of the principal recovery trajectory by a single coefficient (22). This expression coefficient served as correlate to identify the neural pattern, represented as a principal component image of a PCA of the 28 TBM images, most closely associated with recovery. We have shown previously in the context of PET regional cerebral blood flow (CBF) images that PCA provides a powerful tool for elucidating disease-related abnormalities and post-lesional reorganization of neural networks in the human brain (25).

A previous mass-univariate analysis of these TBM images yielded three findings: (i) most striking, impaired patients with chronic disturbed hand motor skills showed the most prominent GMV increase in the ipsilesional medio-dorsal (md) thalamus, including also the head of the caudate nucleus; (ii) all patients evidenced GMV decreases within the contralesional anterior cerebellum at a location typical of cerebellar diaschisis after sensorimotor cortical stroke; and (iii) patients showing fast recovery exhibited a slight GMV increase in the perilesional premotor cortex (PMC). These results stimulated several questions: Does the significant GMV increase of md thalamus in these patients represent an isolated, local effect or does it implicate an extended gray matter network involved in recovery after a sensorimotor cortical stroke? Does the extended network show a structural covariance pattern that discriminates among classes of recovery process? How does the network relate to the initial lesion pattern?

These questions led to the hypotheses examined in the current study: the prominent GMV changes in the md thalamus relate to the dorsolateral prefrontal circuit of Alexander et al. (26) as proposed in our previous paper and may have access to the dysfunctional sensorimotor network post-stroke (22). A posited distributed neuronal network including the md thalamus is specifically related to sensorimotor hand skill. This network manifests a structural covariance pattern that may distinguish among patient subgroups according to recovery class. The structural covariance pattern shows a correlation with the initial lesion pattern.

## Participants and Methods

### Patients and healthy controls

We prospectively recruited patients at two comprehensive stroke centers (Departments of Neurology, University Hospital Bern and Kantonsspital St. Gallen, Switzerland) from January 01, 2008 through July 31, 2010. Inclusion criteria were (1) first-ever stroke, (2) clinically significant contralesional sensorimotor hand function impairment as leading symptom, and (3) inclusion of the pre- and/or post-central gyri within the ischemic lesion confirmed on acute diffusion-weighted (DWI) and fluid attenuated inversion recovery (FLAIR) MRI scans. Patients were excluded if they presented (1) aphasia or cognitive deficits that precluded understanding the study purposes or task instructions, (2) prior cerebrovascular events, (3) occlusion or stenosis >70% of the carotid arteries in MR–angiography, (4) purely subcortical stroke, and (5) other medical conditions interfering with task performance. We recruited 36 patients, seven of which dropped out (three withdrew consent, two were too frail for repeated testing, one was shown to have no cortical stroke after enrollment, one was lost to follow-up). The final sample consisted of 29 patients (five female). As a control group for the analyses of behavioral and clinical data, we recruited 22 healthy older adults (11 female) from the local community. Groups were matched for age (unpaired two-tailed *t*-test: *t*(49) = 3.4, *p* < 0.12) and handedness according to the Edinburgh Handedness Questionnaire (unpaired two-tailed *t*-test: *t*(49) = 0.36, *p* < 0.30). The study received ethical approval from both research centers [Ethikkommission des Kantons St. Gallen (EKSG), Kantonsspital St. Gallen, 9007 St. Gallen and Kantonale Ethikkommission Bern (KEK), 3010 Bern, Switzerland]. All participants gave written informed consent before enrollment according to the Declaration of Helsinki. The same cohort was used for our previous publications (21, 22, 27).

### Data acquisition

#### Study Timeline

We performed a baseline examination within the first 2 weeks after stroke (median 5 days, range 1–18 days) with extended measurements of clinical and behavioral data (see below). The same measurements were taken 3 months (91 days, 80–121 days) and 9 months (277 days, 154–303 days) after stroke. During each of these two visits, we acquired high-resolution anatomical imaging data. Patients were additionally seen at monthly intervals in-between these examinations to evaluate recovery of dexterous hand function.

#### Clinical and Behavioral Data

Clinical stroke severity was assessed using the National Institutes of Health Stroke Scale (NIHSS) (28). Hand motor function was assessed with two outcome variables, grip force and dexterity. Grip force was measured by hand dynamometry (HD) with a Jamar Dynamometer (29, 30). Dexterous hand function was measured using the modified Jebsen Taylor Test (JTT), a standardized quantitative assessment that consists of five timed subtests that simulate everyday activities (31). For our current analysis, we relied on data from the JTT subtest “PSO”, which consists of picking six common objects (two paper clips, two bottle caps, two coins) and dropping them into an empty can as fast as possible. As previously shown by our group, PSO explains by far most of the longitudinal variance in JTT scores and allows accurate classification of patient subgroups (see Supplementary Material for details) (21). The two motor tasks measure complementary aspects of hand motor function. Behaviorally, HD is performed with a simple power grip using the whole hand, whereas PSO necessitates precision grip characterized by opposition of the thumb against one or two fingers (32); and furthermore a proper coupling of grasping and lifting phases of objects performing this task which has been shown to be specifically vulnerable in the case of lesioned dorsolateral PMC (33). Neuroanatomically, each grip form is controlled by different components of the sensorimotor network: power grips are mainly controlled by the primary sensorimotor cortices, whereas precision grip control includes the premotor and posterior parietal cortices (34, 35). As a measure of sensorimotor integration, we included a tactile object recognition (TOR) task, which consisted in discriminating 30 everyday objects with either hand (36). This task was administered at the same time as the NIH evaluation. Further details on measurement procedures can be found in the Supplementary Material.

#### Imaging Data

All patients underwent acute phase imaging at admission according to local stroke imaging protocols. This included a diffusion-weighted imaging (DWI) scan and T1-weighted (T1w) anatomical image. At 3 and 9 months after stroke, each patient underwent high-resolution T1w imaging using a 3D-MDEFT with following imaging parameters (37): repetition time TR = 7.92 ms, echo time TE = 2.48 ms, flip angle = 16°, inversion with symmetric timing (inversion time 910 ms), 256 × 224 × 176 matrix points with a non-cubic field of view (FOV) of 256 mm × 224 mm × 176 mm, yielding a nominal isotropic resolution of 1 mm^3^ (i.e., 1 mm × 1 mm × 1 mm), fat saturation, 12 min total acquisition time. Identical prescription of MR images was achieved by use of the Siemens auto-align sequence that automatically sets up consistent slice orientation based on a standard MRI atlas.

### Data analysis

#### Synopsis

Longitudinal clinical and behavioral data were analyzed with a variant of RFA (24). This is a technique that uses summary measures to simplify analysis of serial measurements [cf. Ref. (24) for clinical examples]. As described below and in Ref. (21), we proceed in two levels: at the single-subject level, we summarize each patient’s *z*-transformed longitudinal data using linear and non-linear curve fitting. At the group level, we then calculate a PCA of these curves to derive a number that summarizes each patient’s recovery relative to the whole cohort. The analysis of structural high-resolution imaging data was performed similarly. At the single-subject level, we calculated TBM maps that encode (longitudinal) local GMV change between 3 and 9 months after stroke, as previously described (22). At the group level, we again calculated a PCA to identify regions with co-varying GMV change across time. In analogy to previous work analyzing structural covariance in the human brain, we refer to these maps as *longitudinal structural covariance networks* (38, 39).

#### Response Feature Analysis of Clinical and Behavioral Data

First, each patient’s PSO task data were transformed to *z*-scores using the mean and SD of a healthy control group of 22 age-matched subjects; normal performance was defined as *z* ≤ 0 ± 2.5 units. Then, each patient’s recovery trajectory was identified by fitting a set of linear and exponential models to the *z*-scores, and the best fitting model was selected using Akaike’s information criterion. Patients were classified in three recovery subgroups according to their recovery model: fast (linear recovery trajectory), slow (exponential recovery trajectory converging to *z* ≥ −2.5) and impaired recovery (exponential recovery trajectory converging to *z* < −2.5). The principal component analyses of the PSO and TOR task, and NIH evaluation were performed with Matlab program, *princomp* (The Mathworks, Inc., Natick, MA, USA). The PSO task yielded ten principal component time courses and variances (one per visit); the TOR task and NIH evaluation, three time courses and variances. Each produced 36 patient expression coefficients (or “scores”). The Kaiser–Guttmann criterion was used to select salient principal components (40). Missing data, arising when patient did not show or could not perform task, were replaced by means over all patients at the time point of the missing data; 10 out of 280 planned visits yielded missing data. The present study uses the expression coefficients of the subset of 28 patients for which TBM images were acquired.

#### Lesion Mapping

Lesions were manually traced on DWI images using MRIcron,^1^ as described in Ref. (21). Lesion volumes were calculated by summing all voxels within the resultant binary lesion masks. The latter were used to exclude lesioned voxels during normalization of all images into the stereotaxic Montreal Neurological Institute (MNI) space (see below). Additionally, we built summary lesion maps for each recovery subgroup, which we thresholded at >20% lesion density for comparison with structural data (see below).

#### Tensor-Based Morphometry

Tensor-based morphometry maps were calculated as described in Ref. (22) using SPM8 (version 4667^2^) running on MATLAB (R2009a, MathWorks, Natick, MA, USA). Briefly, we first realigned 3D-MDEFT images from both acquisition time points to correct for position differences. We next used segmentation with cost-function masking to derive gray matter tissue partitions (41, 42). We then calculated in each subject the Jacobian determinants (first derivatives) of high-dimensional deformation fields that transform voxel-by-voxel the T1w image from month 3 onto the T1w image from month 9. Multiplication of the first derivatives with the matter segmentation from month 3 results in a map that encodes matter volume expansion or contraction per voxel across time. These maps were transformed into the stereotaxic MNI space using normalization parameters derived from segmentation. Normalized GMV change maps were finally smoothed with a 12 mm × 12 mm × 12 mm isotropic 3D Gaussian kernel, motivated by previous studies that show a reduction of false positives for this kernel size in voxel-based morphometry studies (43). These smoothed maps were entered in the covariance analysis as described below. Based on our previous study, we used an unbiased region of interest analysis to extract local GMV changes from ipsilesional thalamus, ipsilesional dorsal PMC and contralesional cerebellum (22).

#### Structural Covariance Using Principal Component Analysis

The PCA of the TBM images was performed on a subset of 28 patients representing the volume changes between months 3 and 9 (of the 29 patients retained for the study 1 had to be excluded because of MR motion artifacts). PCA was executed on the images data using in house software written in MATLAB based on the algorithm described by Alexander et al. and Moeller et al. (44, 45). Extracerebral voxels were excluded from the analysis using a mask derived from the gray matter component yielded by segmentation of the anatomical image volume into gray matter, white matter and cerebrospinal fluid followed by the calculation of residual matrices for each of the 28 scans. From matrices whose rows corresponded to the 28 scans and columns to the 132407 relevant voxels in a single image volume were subtracted from each element (i) the mean of voxel values of its column and (ii) the mean of voxel values of its row, and (iii) added to each element the grand mean of all voxel values in the original matrices. The row, column, and grand means of the resulting residual matrices vanish. Using the singular value decomposition implemented in Matlab, each residual matrix was then decomposed into 28 components. Each component consisted of an image volume, i.e., eigenimage, a temporal expression coefficient, i.e., eigenvariate, and an eigenvalue. The squared eigenvalue is proportional to the fraction of variance described by each component; the subject expression coefficients describe the amount that each scan contributes to the component; and the component image displays the degree to which the voxels co-vary in the component in the course from months 3 to 9. The subject expression coefficients and voxel values of a principal component are orthonormal and range between −1 and 1; the orthogonality reflects the lack of statistical correlation among the principal components. Significant clusters were delineated by applying a height threshold at the first and ninety-ninth percentile of voxel values and an extent threshold of 32 voxels (corresponding to the minimal resolution element of the TBM maps). These clusters were localized using the Jülich cytoarchitectonic probabilistic atlas (SPM Anatomy toolbox, Version 1.8, made available through the Human Brain Mapping division at the Forschungszentrum Jülich at http://www.fz-juelich.de/inm/inm-1/DE/Forschung/_docs/SPMAnatomyToolbox/SPMAnatomyToolbox_node.html). Furthermore, we calculated the overlap between each network cluster and subgroup lesion density maps.

### Statistical analysis

We used median and range for descriptive statistics. We first assessed the relationship of structural covariance component expression, clinical and structural variables, e.g., lesion volume and regional GMV change, using Pearson’s correlation coefficient in order to identify the network related to hand function recovery. Next, we assessed differences with respect to subgroups in network expression and behavioral variables. To do so, we first applied the Shapiro–Wilk test and inspected Q–Q plots for each variable to assess deviations from normality. We used then non-parametric tests to compare scalar variables where appropriate, i.e., the Kruskal–Wallis one-way analysis of variance by ranks to assess differences in the central tendency among any of the three subgroups, and the Mann–Whitney *U* test to compare pairs of subgroups against each other. Finally, we used robust (multiple) regression within the framework of the general linear model to test the relationship of network expression, clinical and structural variables to hand function recovery and their interaction across the whole patient cohort. The criterion for significance was set at *p* < 0.05, Bonferroni corrected for multiple comparisons.

## Results

### Clinical and behavioral data

Clinical characteristics of the patient cohort are summarized in Table 1. Representative sections of each subjects’ ischemic lesion can be found in Figure S1 in Supplementary Material. The behavioral data was incorporated in two principal component analyses. The first principal components of PSO and NIH assessments were chosen for further analysis because they explained the greatest fractions of variance, 70 and 90%, respectively, of the corresponding PCAs. RFA of the PSO task indicated that eight patients showed normal motor performance at baseline (subgroup “fast recovery”), ten patients exponential recovery that converged to normal motor performance (“slow recovery”) and eight whose recovery trajectories followed exponential recovery curves that did not reach normal performance (“impaired recovery”) (21).

**Table 1:** **Descriptive statistics of clinical and demographic data of stroke patients at baseline, month 3 and month 9**.

No.	Id	Age	Gender	Side	Etiology	NIH B	NIH M3	NIH M9	mRS B	mRS M3	mRS M9	HD B	HD M3	HD M9	PSO B	PSO M3	PSO M9	TOR B	TOR M3	TOR M9
1	p01	77	M	L	UN	4	2	1	2	1	1	31	40	41	9.7	7.9	5.7	30	30	30
2	p02	50	M	R	OC	7	1	0	4	1	0	6	54	63	0.0	6.0	6.2	25	28	30
3	p03	78	M	R	LAD	5	5	3	3	2	2	15	17	42	13.5	11.1	9.1	28	29	27
4	p05	80	M	L	LAD	2	3	1	2	1	1	42	42	37	10.6	6.5	8.4	30	30	30
5	p06	53	F	R	LAD	6	3	3	3	2	1	11	9	19	29.9	10.1	14.9	0	0	0
6	p07	78	F	R	CE	4	2	2	2	1	1	18	21	21	14.0	7.5	7.1	0	12	24
7	p09	70	F	R	CE	3	2	0	2	1	0	21	31	34	9.1	8.5	6.0	29	30	30
8	p11	41	F	L	LAD	3	2	0	1	0	0	32	37	39	5.6	4.0	5.11	24	30	30
9	p12	54	M	R	UN	4	2	1	3	1	0	14	33	38	8.5	5.5	5.2	30	30	30
10	p15	54	M	L	LAD	6	4	1	3	1	1	10	24	33	38.8	13.1	11.1	0	6	10
11	p16	73	M	R	OC	4	2	0	2	1	0	51	55	55	7.3	4.9	5.3	26	29	30
12	p17	58	M	L	CE	4	2	0	3	0	0	20	39	48	11.5	4.3	4.7	30	29	30
13	p20	70	M	L	CE	6	4	2	3	1	1	24	35	42	12.9	9.7	9.3	0	6	10
14	p24	74	M	R	CE	4	1	0	1	0	0	34	49	50	14.3	6.9	5.1	28	30	30
15	p25	49	M	R	CE	3	2	1	2	1	0	49	59	67	12.3	5.3	5.9	0	6	10
16	p26	44	M	L	CE	3	1	0	1	0	0	9	33	50	11.5	6.0	5.1	30	30	30
17	p30	63	M	L	CE	4	1	1	3	0	0	43	41	45	10.6	6.3	6.3	30	30	30
18	p31	63	M	L	UN	5	0	0	2	0	0	30	48	44	5.3	4.2	4.7	30	30	30
19	p33	75	M	R	LAD	3	2	2	2	1	1	3	14	22	0.0	18.8	11.5	12	28	30
20	p35	78	M	L	LAD	5	3	2	3	1	1	23	48	40	10.1	6.8	6.1	30	30	30
21	p36	60	M	L	CE	4	1	1	3	1	1	31	40	41	18.2	8.0	6.6	30	30	30
22	p37	75	M	R	OC	4	2	1	2	1	1	0	27	32	0.0	8.6	10.4	4	23	25
23	p38	77	M	L	LAD	5	2	2	3	1	1	10	21	23	26.9	10.9	8.3	29	30	30
24	p41	51	M	R	CE	2	1	0	2	1	1	36	41	52	7.1	5.1	4.8	30	30	30
25	p42	64	M	R	LAD	1	0	0	2	0	0	14	33	35	18.9	7.1	7.4	29	30	30
26	p43	82	M	L	LAD	3	3	2	2	2	1	17	10	18	16.8	21.4	13.9	20	22	25
27	p44	67	M	R	UN	11	10	9	4	3	3	15	15	41	52.3	45.1	12.3	3	4	2
28	p45	53	M	R	LAD	11	9	4	5	3	2	0	10	17	0.0	45.5	19.9	0	1	3
Median		65.5	24 M	13 L	11 LAD, 10 CE, 4 UN, 3 OC	4	2	1	2	1	1	20	35	41	11.0	7.3	6.5	28	29	30
Range		41, 82	4 F	15 R		1, 11	0, 10	0, 9	1, 4	0, 3	0, 3	0, 51	9, 59	17, 67	0.0, 52.3	4.0, 45.5	4.7, 19.9	0, 30	0, 30	0, 30
Median (*z*)												−1.3	−0.2	0.4	−5.0	−1.2	−0.4	0.6	0.6	0.6
Range (*z*)												−2.8, 1.3	−2.3, 1.9	1.6, 2.6	−38.9, 0.5	−33.2, 1.6	−11.7, 1.0	−7.5, 0.6	−6.5, 0.6	−4.5, 0.6

*M, male; F, female. Etiology is classified according to the Trial of ORG 10172 in acute stroke treatment (TOAST): LAD, large artery disease; CE, cardioembolism; OC, other determined cause; UN, undetermined cause. NIH, National Institutes of Health Stroke Scale; mRS, modified Rankin Scale; HD, hand dynamometry (in kilograms); PSO, picking small objects task (in seconds); TOR, tactile object recognition (in numbers of recognized objects); *z*, *z*-scores using mean and standard deviation of healthy controls for each task*.

### Selection of longitudinal structural covariance networks

Table 2 characterizes three principal components of the TBM images (structural covariance networks) that correlated with clinical and behavioral variables across the whole patient cohort. The first component (PC1_TBM_) correlated with GMV reduction in the cerebellum contralateral to the affected hemisphere. The second component (PC2_TBM_) correlated with lesion size, GMV volume increase in the md thalamus, clinical (PC1_NIHSS_ expression) and hand function specific recovery (PC1_PSO_ expression). The fourth principal component correlated exclusively with PC1_NIHSS_ expression. None of the other PCs surviving the Kaiser–Guttmann criterion correlated with any of the external variables.

**Table 2:** **Correlation of longitudinal structural covariance networks across all patients (*n* = 28)**.

Component	Variance (%)	Parameters with significant correlations^a^	Values of parameters^b^	Correlation coefficient (*r*)
PC1_TBM_	19.9	GMV change ant. cerebellum	−0.2 (−1.3, 0.6) %	−0.57
PC2_TBM_	9.1	Lesion volume	9.0 (0.6, 141.7) cc	0.61
		PC1_NIHSS_ expression	−3.65 to 11.9	0.61
		PC1_PSO_ expression	−20.99 to 64.26	0.51
		GMV change md Thalamus	0.4 (−0.6, 4.0) cc	0.72
PC4_TBM_	8.1	PC1_NIHSS_ expression	0 (−3.65, 11.9)	0.54
Cumulative Variance	37.1			

*NIHSS score, National Institute of Health Stroke Scale-score; PSO, picking small objects, MI primary motor cortex, SI primary sensory cortex*.

*^a^Significant correlations after correction for eight multiple comparisons: 0.05/8 = 0.006 yields significant entries. This probability corresponds to a correlation coefficient of 0.466*.

*^b^Values of parameters are indicated as median, including range, expression coefficients are indicated as range due to normalization (median of 0)*.

### Thalamocortical network related to hand function recovery

#### Effects Across the Patient Cohort

**Figure 1:**
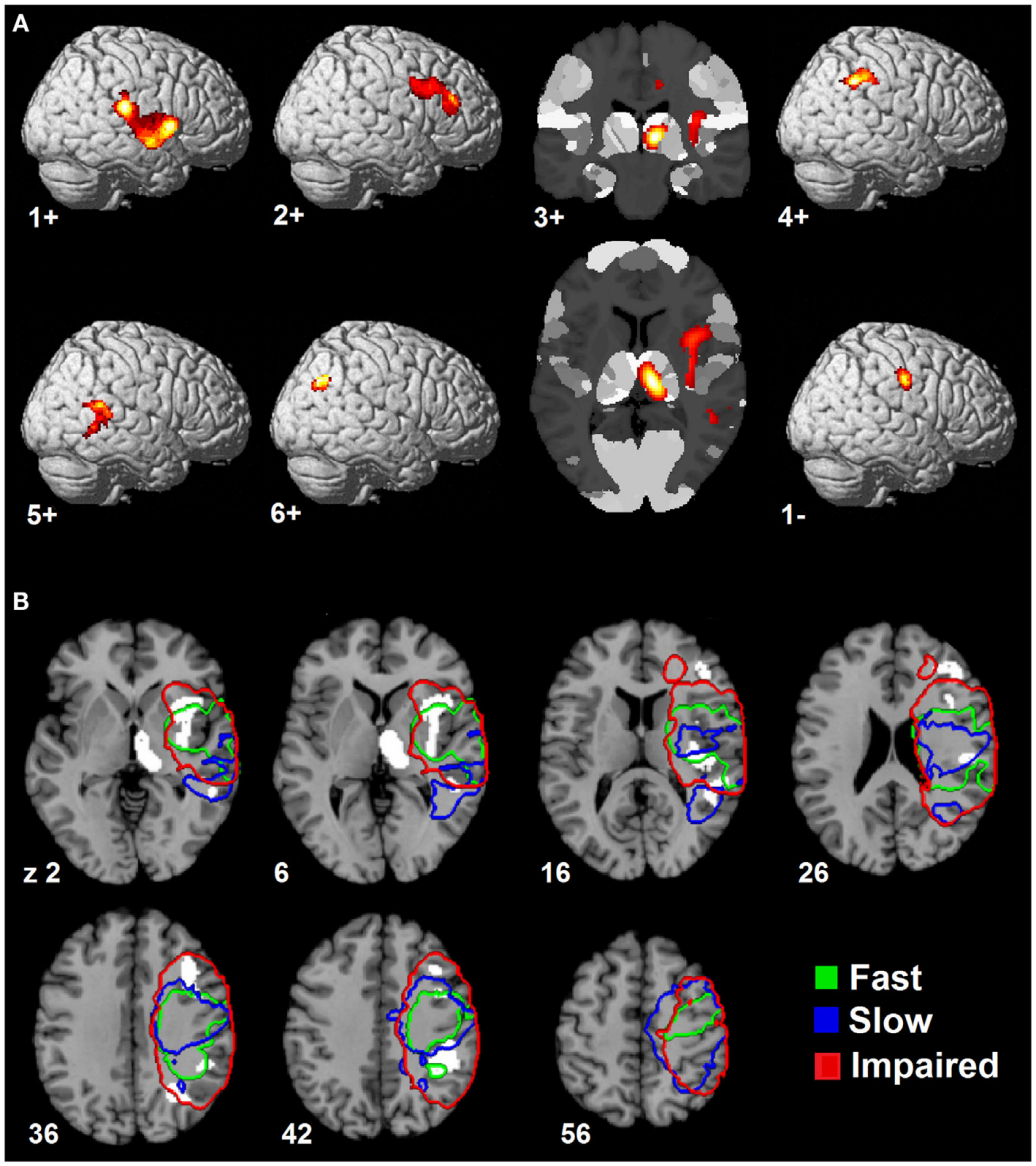
**Spatial topography of longitudinal structural covariance network correlating with hand function recovery**. **(A)** shows the six largest clusters of supra-threshold voxels for the second principal component (PC2_TBM_) projected onto a standard three dimensional brain and onto a cytoarchitectonic atlas (cluster 3+) in MNI space. Clusters are labeled according to their (positive or negative) correlation with gray matter volume expansion in the medio-dorsal thalamus. The threshold for positive clusters corresponds to the first percentile of voxel values (absolute value 0.0064), the threshold for the negative cluster to the ninety-ninth percentile (absolute value 0.0095). **(B)** shows the spatial relationship between the covariance network clusters and lesion maps of patient subgroups. Color-coded contours define areas with ≥20% lesion probability in each subgroup. Size, localization, cytoarchitectonic assignment, and functional correlates of the individual clusters are summarized in Table 3.

**Figure 2:**
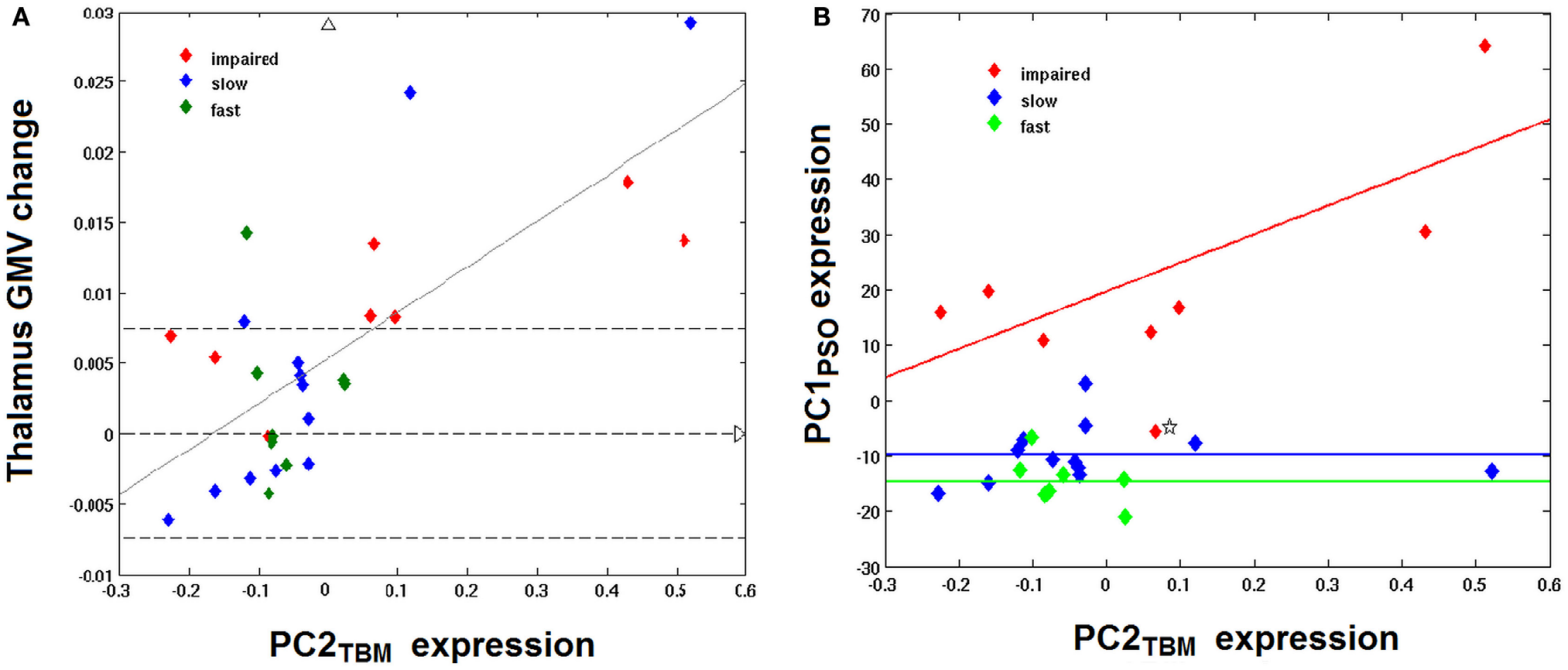
**Correlation between PC2_TBM_, thalamic GMV change, and longitudinal hand function recovery**. **(A)** shows the relationship between thalamic gray matter volume (GMV) change and expression of the structural covariance network PC2_TBM_: thalamus GMV change = 0.0325 × PC2_TBM_ + 0.0053; *R* = 0.72, *p* < 0.001. Dashed transversal lines indicate the interval of reliable GMV change (± 0.75%), as determined in previous studies. **(B)** shows the correlation between expression of the structural covariance network PC2_TBM_ and the first component of longitudinal behavioral recovery of skilled hand function, PC1_PSO_. Only impaired patients show a strong correlation between network expression and hand function recovery; PC1_PSO_ = 51.9 × PC2_TBM_ + 19.76; adjusted *R* = 0.74, *p* < 0.05. The recovered subgroups are characterized by a constant of differing magnitude. One patient with negative expression coefficients of PC1_PSO_ (marked by an asterisk) has been identified as outlier (see text).

Since the second structural covariance network PC2_TBM_ correlated with our specific measure of hand function recovery, we focused further analysis on its critical clusters (or nodes, Figure 1A). Clusters that co-varied with the thalamus fell within the first percentile of voxel values, and were labeled as “positive” clusters since the thalamus showed gray matter increase. These clusters (ordered by size) included insular and peri-insular cortex, dorsolateral prefrontal and ventral premotor cortices, thalamus, posterior parietal cortices and two smaller clusters in the temporal and occipital cortex. A single cluster fell within the ninety-ninth percentile and included pre- and post-central cortex. Table 3 summarizes localization, statistics and functional correlates of all clusters that survived thresholding (PC1_TBM_ and PC4_TBM_, are summarized in Tables S1 and S2 in Supplementary Material, respectively). Functional interpretation was done in the context of motor hand function, based on current literature. The expression of this network had a strong correlation with thalamic GMV change across the whole cohort (Figure 2A).

**Table 3:** **Clusters of the longitudinal structural covariance network (PC2_TBM_) related to hand function recovery: size, localization, cytoarchitectonic assignment, and functional correlates**.

Cluster	Size (*n* vox.)	MNI (max.)	Anatomical area	Cytoarchitectonic area	Functional correlate (references in brackets)
**First-percentile voxels (height threshold: 0.0064, extension threshold: 32 voxels)**					
1+	1362	38/−28/16	R. parietal operculum	OP1, OP2, OP3	Tactile working memory, stimulus discrimination and perceptual learning (41–44)
			R. insula	Ig1, Ig2	Multisensory processing (36, 50–53)
		54/−26/28	R. inferior parietal lobule	PFcm, PFop, PFt	Action observation and imitation (47–49)
2+	653	43/26/26 40/10/34	R. DLPFC (dorsal-posterior part) R. ventral premotor cortex	n.a. n.a.	Action execution and working memory (34, 35) Motor hand skill related to intrinsic objects properties (83)
3+	502	10/−20/6	R. thalamus	Thal: prefontal Thal: temporal Thal: parietal	MD nucleus to prefrontal cortex (33–35) MD nucleus to temporal lobe (33–35) LP/Pu complex to parietal lobe (33–35)
4+	408	42/−38/42	R. intraparietal sulcus R. post-central gyrus R. inferior parietal lobule	hIp1, hIp2, hIp3 BA2 PFt, PFm	Spatial attention, visuomotor transformation (57, 66–68) Primary somatosensory information processing (56) For PFt see above; for PFm non-spatial attention (49)
5+	271	52/−48/2	R. superior (and middle temporal) gyrus	n.a.	Spatial awareness (69)
6+	158	30/−62/36	R. middle occipital gyrus	n.a.	Spatial processing of tactile stimuli (70)
**Ninety-ninth-percentile voxels (height threshold: 0.0095, extension threshold: 32 voxels)**					
1−	179	54/−14/38	Pre- and post-central gyrus	BA 4p, 3b, 1, 2	Voluntary and passive finger motion (BA 4p) (71) Somatosensory information perception (3b) and processing (1, 2, 73)

#### Effects Within Patient Subgroups

Having identified a structural network related to hand function recovery (Table 4), we next analyzed its relationship to lesion topography within recovery subgroups. Lesion analyses are summarized in Figure 1B. Projection of subgroup lesion density maps onto PC2_TBM_ clusters showed that the thalamic cluster was spared across all subgroups, but that the other clusters showed varied involvement. A detailed volumetric analysis (Table 5) showed that only a small fraction of each lesion density map affected network clusters (median and range 0.95%, 0–6.7%), indicating that GMV density changes occurred either in perilesional or more distant areas. When analyzing the percentage of each cluster affected by the lesion, there were notable differences: lesions in the fast recovery subgroup affected mostly the parietal-opercular and insular cluster (Cluster 1+), whereas lesions in the impaired subgroup affected mostly the ventral premotor cortex and intraparietal sulcus (IPS) (Cluster 3+ and 4+). The slow recovery subgroup showed no clear lesion profile. Affection of the pre/post-central cluster (Cluster 1−) increased across subgroups.

**Table 4:** **Clinical and structural variables across recovery subgroups**.

	Fast recovery *n* = 8	Slow recovery *n* = 12	Impaired recovery *n* = 8	Kruskal–Wallis, *p*	Mann–Whitney, impaired versus recovered *p* (2-tailed)
**Network of gmv change between months 3 and 9**					
PC2_TBM_ expression coeff.	−0.079 (−0.117, 0.025)	−0.04 (−0.23, 0.52)	0.06 (−0.22, 0.51)	0.55	n.a.
**Parameters tested for correlation**					
Age	63 (41, 73)	75 (49, 80)	68.5 (53, 82)	0.31	n.a.
Lesion size (cc)^a^	7.80 (0.76, 75.52)	3.48 (0.57, 70.39)	42.84 (2.72, 141.71)	0.08	<0.05
PC1 (NIH) expression coeff.^a^	−2.18 (−3.07, 0.61)	−1.31 (−3.65, 2.12)	1.33 (−1.40, 11.90)	<0.01	<0.01
PC1 (PSO) expression coeff.^a^	−15.3 (−21.0, 6.7)	−10.8 (−16.8, 3.1)	16.5 (−5.6, 64.3)	<0.0001	<0.0001
PC1 (TOR) expression coeff.	13.9 (−0.45, 14.3)	13.9 (11.0, 14.3)	−29.3 (−34.3, 13.7)	<0.001	<0.001
GMV premotor area	0.0043 (−0.0010, 0.0092)	0.0017 (−0.0013, 0.0078)	0.0011 (−0.0020, 0.0083)	0.69	n.a.
GMV thalamus^a^	0.0017 (−0.0043, 0.0143)	0.0023 (−0.0061, 0.0292)	0.0083 (−0.0002, 0.0179)	0.06	<0.05
GMV cerebellum	−0.0019 (−0.0130, 0.0031)	−0.0029 (−0.0089, 0.0060)	−0.0012 (−0.0121, 0.0051)	0.43	n.a.

*All values are given as median (range)*.

*NIH, National Institutes of Health Stroke Scale; PSO, picking small objects; PC1, first principal component of longitudinal data of corresponding clinical or behavioral variable; PC2_TBM_, second principal component of tensor-based morphometry data; GMV, gray matter volume*.

*Significant correlations after correction for multiple comparisons: at a nominal alpha level of 0.05 and eight correlations, a p-value of 0.05/8 = 0.006 yields significant entries. This probability corresponds to a correlation coefficient of 0.466*.

**Table 5:** **Overlap between subgroup lesion density maps and longitudinal structural covariance network related to hand function recovery**.

		Cluster 1+	Cluster 2+	Cluster 3+	Cluster 4+	Cluster 5+	Cluster 6+	Cluster 1−
		pOP	vPMC	Thal	IPS	STG	MOG	PCG
*Raw volume (cc)*		10.9	5.2	4.0	3.3	2.2	1.3	1.4
Fast	105.7	7.10	0.3	0	1.0	0.3	0	0.3
Slow	113.9	1.82	0.5	0	1.0	1.0	0.2	0.6
Impaired	239.7	8.7	3.3	0	3.3	1.1	0.9	1.4
*Percent of lesion on cluster*								
Fast		6.7	0.3	0	0.9	0.3	0	0.3
Slow		1.6	0.4	0	0.9	0.9	0.2	0.5
Impaired		3.6	1.4	0	1.4	0.5	0.4	0.6
*Percent of cluster affected*								
Fast		61.5	5.8	0	30.3	13.6	0	21.4
Slow		14.7	9.4	0	29.7	46.4	17.7	44.3
Impaired		33.0	62.7	0	98.8	51.4	69.2	100.0

*Cluster labels correspond to Table 3, additionally including main anatomical region within each cluster. Volumes are calculated for subgroup density maps in Figure 1B and each cluster separately*.

*pOP, parietal operculum; vPMC, ventral premotor cortex; Thal, thalamus; IPS, inferior parietal sulcus; STG, superior temporal gyrus; MOG, middle occipital gyrus; PCG, pre- and post-central gyri*.

We further compared the patients subgroups presenting normal motor performance after 9 months (fast and slow recovery) with the subgroup that did not achieve normal performance (impaired recovery). As expected from the RFA, the latter group yielded the highest expression coefficients in PC1_NIHSS_ (*p* < 0.01) and specifically in PC1_PSO_ (*p* < 0.0001). This group had also the largest GMV expansion in the medio-dorsal thalamus and the highest lesion volumes (both *p* < 0.05). Figure 2 shows the relationship between PC2_TBM_ expression and thalamic GMV change (panel A) and hand skill recovery as reflected by PSO (panel B), respectively. Considering all individuals, GMV change correlated with expression coefficients of the structural covariance network of PC2_TBM_ (*R* = 0.72 and *p* < 0.5 after correction for multiple comparisons). PC2_TBM_ expression could also distinguish between subgroups: When dividing patients into subgroups with positive versus negative network expression coefficients (without regard to recovery subgroup assignments), we found that the positive subgroup has significantly higher thalamus GMV change (median 1.35% with range 0.83–1.79%), whereas the negative subgroup shows no significant change (median 0% with range 0.04–0.04%, Mann–Whitney *U* test *p* < 0.001).

However, only the impaired recovery subgroup showed a linear relationship between the expression of structural covariance network of PC2_TBM_ and recovery (Figure 2B): the slope estimate (and SE) was 53.1 ± 22.7; adjusted *R* = 0.74 with *p* < 0.05. Note that one patient of this subgroup showed a negative PC1_PSO_ expression score. Inspection of the raw data indicated that this particular subject showed a secondary deterioration of skilled hand function during the last 2 months of the study, after an initially favorable course. Removal of this outlier did not change results. A few individuals of the recovered subgroups exhibited high GMV changes in the medio-dorsal thalamus, representing exceptions to the group trend.

#### Multivariate Linear Regression

To further test the specificity of the association between PC2_TBM_ and hand function recovery, we calculated a multivariate linear regression of PC1_PSO_ onto covariance network expression, age, volume, and thalamic GMV change: it showed significant effects of the model intercept (*p* = 0.036), PC2_TBM_ expression (*p* = 0.048) and lesion volume (*p* = 0.037). The significant intercept indicated residual variance not modeled by our predictors. We, therefore, investigated a reduced model that included PC1_PSO_ as dependent variable, and only the significant predictors from the first model, i.e., PC2_TBM_ expression, lesion volume and their interaction (PC2_TBM_ expression × lesion volume) as independent variables. The interaction term significantly predicted PC1_PSO_ scores (β = 1.1, *t*(24) = 3.83, *p* < 0.001) over and above the other variables (both *p* > 0.1). The interaction term explained a significant portion of variance in hand function recovery (*R*^2^ = 0.639, *F*(3, 24) = 14.18, *p* < 1.6e−5). Full model parameters are summarized in the Table S3 in Supplementary Material.

## Discussion

In this study, we have identified structural covariance networks deduced from GMV changes during the recovery of patients suffering from hand paresis after ischemic sensorimotor stroke. These networks correspond to the first, second, and fourth principal components determined from a PCA of TBM images and explained 19.9, 9.1, and 8.1% of the variance, respectively. Implied by the correlation of its expression coefficients with GMV-decrease in the anterior cerebellum contralateral to pre- and post-central infarction in all patients, the first component PC1_TBM_ appears to reflect a neuronal network caused by diaschisis from sensorimotor cortex (46). The second component PC2_TBM_, associated with a specific manual skill, i.e., precision grip, as implied by its correlation with PC1_PSO_ represents a neuronal network involving GMV- increase in the md thalamus. Finally, the correlation of the fourth component expression coefficients with the NIHSS scores summarized in PC1_NIHSS_ suggests that the corresponding network reflects general neurological deficit. A third behavioral parameter of sensory information processing, TOR, showed no significant correlation with a principal component, although a deficit persisted in the impaired subgroup.

Finally, a multivariate linear regression approach verified (i) the unique relationship of PC1_PSO_ to the structural covariance network of PC2_TBM_; and furthermore, that this relationship is related specifically to the network expression but not to a single constituent, e.g., md thalamus. Since PC2_TBM_ relates directly to hand function recovery and thus to our study aim, we will discuss this network in more detail in the following.

### Associations of the structural covariance network with external variables

This study represents important progress following our recent paper on “Gray matter volumetric changes related to recovery from hand paresis after cortical sensorimotor stroke” (9) as it relates the most prominent finding of gray matter increase in the md thalamus in patients after a first-ever stroke to a large distributed structural covariance network including a cortico-striato-thalamic loop and diverse sensorimotor cortices.

Irrespective of the clinical and behavioral course, this PC2_TBM_ network distinguishes clearly within the study cohort since the subgroup with positive expression coefficients is associated with large GMV increases in the md thalamus between months 3 and 9, while the subgroup with negative expression coefficients did not exhibit a recognizable GMV change. The GMV increases in the former subgroup exceed the measurement uncertainty and are consistent with the few comparable studies, e.g., in the paper of Gauthier et al. (32). As Table 2 shows, the neural network represented by PC2_TBM_ is significantly related to the recovery of motor hand skill in the patient cohort; however, only the impaired recovery subgroup shows a strong linear regression, while the fast and slow recovery groups show little correlation with PC1_PSO_ (Figure 2B). A multivariate linear regression positing the dependence of PC1_PSO_ on the three salient principal components as well as on age, lesion volume, and GMV change in the thalamus showed significant effects only in PC2_TBM_ and lesion volume. A refined analysis showed a significant interaction between these two variables, and revealed that the interaction was the only significant explanatory variable. The fast and slow recovery groups indicated an inverse relationship between PC2_TBM_ and lesion volume; the greater lesion volumes were accompanied by smaller component expression coefficients, and vice versa. In contrast, the members of the impaired group exhibiting the largest interaction expressed most strongly PC1_PSO_.

### Network topography and suggested functions

The salient regions of the second principal component PC2_TBM_ are summarized in Table 3; the regions characterized by voxel intensities of the first percentile contain the thalamic cluster. Using a probabilistic atlas of white matter connections, we found that this thalamic cluster was located on regions of the md thalamus that are preferentially connected to prefrontal, temporal and parietal cortex (33, 34). These three cortical regions were also found in the set of regions belonging to the first percentile, underscoring the importance of the thalamic gray matter increase. The implicated md thalamus and dorsolateral prefrontal cortex are constituents of the subcortico-cortical, dorsolateral prefrontal loop (35). The involvement of this dorsolateral prefrontal-striato-thalamic loop suggests a compensatory mechanism to maintain motor execution by cognitive control once the primary (more automatic) sensorimotor network of hand motor skill is dysfunctional (47).

Both parts of posterior medial thalamus and dorsal-posterior subarea of the dorsolateral prefrontal cortex are interconnected with the posterior parietal cortex (PPC) (48), which our previous VLSM studies (8) have shown to be seriously affected in the impaired subgroup.

Densely interconnected structures of ventral PMC, PPC, SII and posterior insula are represented in the component image of PC2_TBM_, representing possible sub-networks engaged in higher order sensorimotor information processing and spatial awareness (see below). In the PPC locally functional processed information, e.g., space and action perception, is transmitted via feedback loops to ventral PMC (34, 49, 50). The areas co-varying positively with the thalamus represent a complex neuronal network consisting of functional and dysfunctional nodes. The functional nodes outside of the lesions comprise the dorsolateral prefrontal loop for motor execution (26), whereas the dysfunctional nodes include various higher order sensorimotor cortices within the lesions. Performance of sensorimotor hand skill, especially in the impaired recovery group, is related to lesion size and extension into network nodes in ventral PMC, PPC, SII, and posterior insula.

A remarkable feature of the structural covariance pattern is the appearance of the parietal operculum subarea OP1 in the absence of OP4. OP4 plays a role mainly in basal sensorimotor integration processes, e.g., incorporating sensory feedback into motor actions which are the basis for information processing during tactile exploration (51, 52). The involved OP1 seems to support more complex information processing demanded during tactile working memory, stimulus discrimination, and perceptual learning (53–56). These differing functional roles are reflected by the distinct connectivity profiles of the areas: OP4 is connected to fronto-parietal areas, while OP1 is connected predominantly to the inferior parietal cortex (IPC) (57). In a three-region model in humans, the rostral IPC, including PFcm, PFop, PFt, has been shown to be involved in reaching and grasping (58). The very rostral part (PFop) seems to be activated specifically during observation of tool use. Moreover, meta-analyses indicated the participation of PFt in action observation and imitation networks (59–61). In humans somatosensory activation of the posterior insula has been observed during simple stimulation paradigms, e.g., estimation of the roughness of gratings and TOR, suggesting a role in somatosensory processing (62–65). Multisensory processing in the posterior insula has also been observed in primate experiments with responses also to auditory, baroreceptive and painful stimuli (66, 67).

As has been shown in primates, while area 2 is activated by fine grained proprioceptive sensory information obtained by transitive finger movements (68), specific neuron populations within anterior IPS (AIP) are activated by grasping and manipulation of 3-D objects as well as by visual fixation of objects (69). Analogously, in humans area 2 is involved in the perception of geometrical and texture characteristics like edge length and roughness. This function contrasts to the putative human homologue of the IPS, which responds to shape perception, including somatosensory discrimination, visuo-tactile matching, and, together with premotor cortices, skilled motor manipulation of 3-D objects (50, 70–73). The human IPS has been characterized using cytoarchitectonical techniques (74, 75). Functional connectivity analyses have shown these sub-areas along the IPS to be distinguished by distinct connections (76). The AIP ROIs (hIP1 and hIP2) connect mainly to frontal attentional regions, whereas posterior IPS (hIP3) connects mainly to posterior occipital regions. Analog connections have been shown in macaque anatomical studies, e.g., the strong connections between the AIP and ventral PMC and the posterior IPS (CIP) to visual cortices (77). This explains also visuomotor coordination via the AIP and the implication of the posterior IPS in peripersonal visual representations (78–80). Karnath et al. found that in patients free of lesions in visual as well as subcortical structures, the critical site for spatial awareness was located in the superior temporal gyrus (BA 22 and 42) (81). Using fMRI it could be shown that the right middle occipital gyrus processes spatial rather than non-spatial auditory and tactile stimuli (82). In a review, Rizzolatti et al. conclude that the ventral PMC executes both motor and cognitive functions: motor functions comprise hand actions related to intrinsic object properties and head and arm actions related to spatial locations, whereas cognitive functions include space perception, action understanding and imitation (83). In the context of our study the observation of Ehrsson et al. is of importance as they found that precision grip showed more extending activations compared to power grip, involving ventral PMC in both hemispheres (35).

Of the salient regions of the second principal component PC2_TBM_, a single cortical cluster contains voxels belonging to the ninety-ninth percentile, which presumably characterizes fast and slow recovered individuals. It includes a sub-network within pre- and post-central gyrus, ventral to the center of gravity of the lesion in the slowly recovering subjects as described in our previous paper (21). The isolated involvement of 4p, but not of 4a, substantiates the double representation of the motor system in the precentral gyrus, the former activated in simple motor tasks, whereas the latter responds to more complex and self-initiated tasks (84). In activation studies of healthy individuals, voluntary and passive finger motion stimulated areas 4p and 3a, simple sensory stimulation areas 3b, 1 and 2 and complex sensory stimulation area 4a (85).

### Limitations

This study comprises a detailed evaluation and discussion of structural covariance networks associated with hand motor skill. At the outset, the number and composition of recovery subgroups in the patient cohort was unknown. Thus, the number of patients in each subgroup is relatively small. Larger cohorts would be desirable to assign subjects reliably to subgroups characterized by distinct patterns of structural reorganization associated with varying degrees of recovery. Besides subgroup specific patterns, especially in the subgroup with slow but complete recovery, the assessment of idiosyncratic aspects, e.g., exceptions to the involvement of the dorsolateral prefrontal-striato-thalamic loop, is another challenge. Meeting it would necessitate detailed protocols, including a comprehensive neuro-rehabilitation program, reporting of targeting interventions and physiological measures of movement efforts versus efficiency of motor activity. As the existence of the subgroup with fast complete recovery indicates, an earlier begins after stroke of the study might help to assess structural plasticity in the first 3 months when most recovery occurs. The incomplete gender matching must also been taken into consideration, because women have been shown to perform dexterity tasks (nine-hole peg test) faster than men depending on age, and upper limb kinesthetic asymmetries in contralateral reproduction of elbow movements, elicited by tendon vibration, were prevalent in males (86, 87).

## Conclusion

As posited in Section “Introduction”, our study confirms that the md thalamus, distinguished by significant gray matter increase after first-ever stroke, is a constituent of an extensive structural covariance network encompassing (i) a cortico-striato-thalamic loop involved in motor execution and (ii) higher order sensorimotor cortices affected to varying degrees in the study cohort. Positive expression coefficients of the network are associated with significant GMV increases in the md thalamus in contrast to negative expression coefficients. This brain structural covariance pattern reflects a specific structural covariance network related to recovery of motor hand skill and may distinguish among patient subgroups according to recovery class. The surrogate marker for motor hand skill, PSO, depends on an interaction between the expression of the network and lesion volume. Related to this condition, the impaired group exhibiting the largest interaction expressed most strong PC1_PSO_ and inversely were limited in the expression of the structural covariance network of PC2_TBM_. To conclude, our application of tensor-based morphology has shown it to be a powerful method for studying gray matter changes after stroke; it is capable of revealing both local changes and in associated extensive neural networks. Regarding its future use application, TBM will be potentially of interest in the study of targeted treatment effects in the long-term.

## Conflict of Interest Statement

The authors declare that the research was conducted in the absence of any commercial or financial relationships that could be construed as a potential conflict of interest.

## Supplementary Material

The Supplementary Material for this article can be found online at http://journal.frontiersin.org/article/10.3389/fneur.2015.00211

## Footnotes

^1^https://www.nitrc.org/projects/mricron

^2^http://www.fil.ion.ucl.ac.uk/spm/software/spm8/
