# Supplementary material for: A Thalamic-Fronto-Parietal Structural Covariance Network Emerging in the Course of Recovery from Hand Paresis after Ischemic Stroke
Source: Front Neurol. 2015 Oct 13;6:211. doi: 10.3389/fneur.2015.00211 (PMC4602136; doi:10.3389/fneur.2015.00211)
Supplement: Supplementary file 1 [file Presentation_1.PDF]

A thalamic-fronto-parietal structural covariance network emerging in the course of recovery from hand paresis after ischemic stroke

Eugenio Abela[1], John Missimer[2], Andrea Federspiel[3], Andrea Seiler[1, 4], Christian Walter Hess[4], Matthias Sturzenegger[4], Roland Wiest[1] and Bruno Weder[1, 5*]

1. Support Center for Advanced Neuroimaging (SCAN), Institute for Diagnostic and Interventional Neuroradiology, University Hospital Inselspital and University of Bern, Switzerland
2. Paul Scherrer Institute, Laboratory of Biomolecular Research, Villigen, Switzerland
3. Department of Psychiatric Neurophysiology, University Hospital of Psychiatry and University of Bern, Switzerland
4. Department of Neurology, University Hospital Inselspital and University of Bern, Switzerland
5. Department of Neurology, Kantonsspital St. Gallen, Switzerland

Supplemental materials and methods

Behavioral Assessment

All behavioral measurements were made by the same author (EA).

Hand dynamometry

A Jamar dynamometer was used to measure maximal hand dynamometry (HD) over three trials, alternating between hands [1]. The dynamometer was held in front of the subject with the elbow flexed at 90° and slightly abducted to avoid contact with the trunk. Subjects were instructed to squeeze the lever of the apparatus as strongly as possible without extending the arm. An investigator supported the dynamometer such that the participant could comfortably exert a power grip without the need to additionally stabilize the dynamometer. Values were recorded in kg.

Dexterous hand function

Dexterity was measured for both hands using the modified Jebsen-Taylor Test (mJTT), a standardized quantitative assessment of hand function that consists of five timed subtests intended to simulate everyday activities, e.g. turning pages, eating using a spoon [2]. The test is scored by adding the seconds needed to complete each subtest (higher values indicate worse performance); standard performance norms are available from the original publication for both sexes and for different age groups [2]. Several studies have used this test to assess hand function in stroke [3-6]. We applied the mJTT with custom-made materials according to the original instructions. Specifically, for the Picking Small Objects (PSO) task, two paper clips, two bottle caps and two coins were positioned on the same side as the tested hand, touching a wooden board. The subject had to pick each object, starting with the one farthest away and put them into a can positioned at the body midline. The task was performed in mirror symmetric fashion for both the contra- and ipsilesional hand.

Task selection

Although the mJTT is originally scored by summing its subtests, not all of them might be equally sensitive to longitudinal change. To analyze the response characteristics of each tasks, we calculated the longitudinal variance of each task across time, and the average within and between-subjects variance [10]. In order for this paper to be self-contained, we repeat our main results of this analysis from Ref. 10. in the supplementary results section. Additionally, we analyzed whether keeping only the PSO subtest for longitudinal principal component analysis (PCA, see main text) would lead us to miss relevant variance associated with other motor functions (and thus potentially reflecting recovery with different neural substrates). To this end, we calculated first longitudinal PCA for each of the subtests left out (Stacking Checkers, Turning Cards, Lifting Light Cans, Lifting Heavy Cans) and then computed the correlation coefficient between each first principal component (PC) of these tasks and the first PC of PSO. We expected high correlations to indicate that PSO indeed captured all the behavioral relevant variance in mJTT performance.

Imaging Data Preprocessing

Anatomy: Normalization and Cost-Function Masking

Anatomical data were normalized using SPM8 (http://www.fil.ion.ucl.ac.uk/spm/software/) for MATLAB. The SPM8 framework uses a set of affine (linear) and non-linear transforms to

match an individual image (e.g. T1 scan) to a stereotactic template. Mismatch of images is quantified as the sum of the squared differences between the image voxel intensities (i.e. a "cost function") that is reduced during image registration [7]. While the affine part is relative insensitive to the presence of focal lesions, the non-linear estimation of the algorithm can lead to distortion of the damaged area and the surrounding tissue, usually resulting in reduced lesion volumes [7, 8]. This remains true for most real-world scenarios, even if newer normalization and registration algorithms perform well with simulated lesions [9]. It is therefore recommended to exclude the damaged voxels during the normalization process, in order to reduce their impact on cost function minimization [7]. To accomplish this, we used a "cost function masking" procedure with explicit binary lesion masks, as previously described [10]. First, all lesions were manually segmented by one author (EA) in native space onto diffusion weighted images (DWI) acquired at the acute phase (baseline) using MRIcron (http://www.cabiatl.com/mricro/mricron/index.html), yielding binary lesion images. The advantage of using baseline DWI scans is twofold: they provide superior contrast for the identification of the acute ischemic lesion (compared to T1 or T2-weigthed images), and acute DWI lesion volumes have an excellent correlation with chronic stroke lesion volumes [11]. To avoid bias, lesion segmentation was performed without reference to the results of the behavioral or CBF data analysis. Lesion images were smoothed with a 3D Gaussian kernel with 8.0 mm full-width at half maximum, inverted and binarized at a threshold of 0.1 to yield the final lesion masks [7]. DWI scans and lesion masks were then co-registered to the T1-images from Month 3. Finally, co-registered lesion masks and T1-images were simultaneously spatially normalized to Montreal Neurological Institute (MNI) stereotaxic space using the unified segmentation algorithm in SPM8 and resampled to 2.0 mm3 isotropic resolution.

Supplemental results

Task Selection

The bar chart bellow summarizes the longitudinal cumulative (CumVar) and task variance between stroke patients. As can be seen from the chart, the task Picking Small Objects (PSO, Pick), had the largest variance of all subtests across the observation period.

Longitudinal between-subjects variance

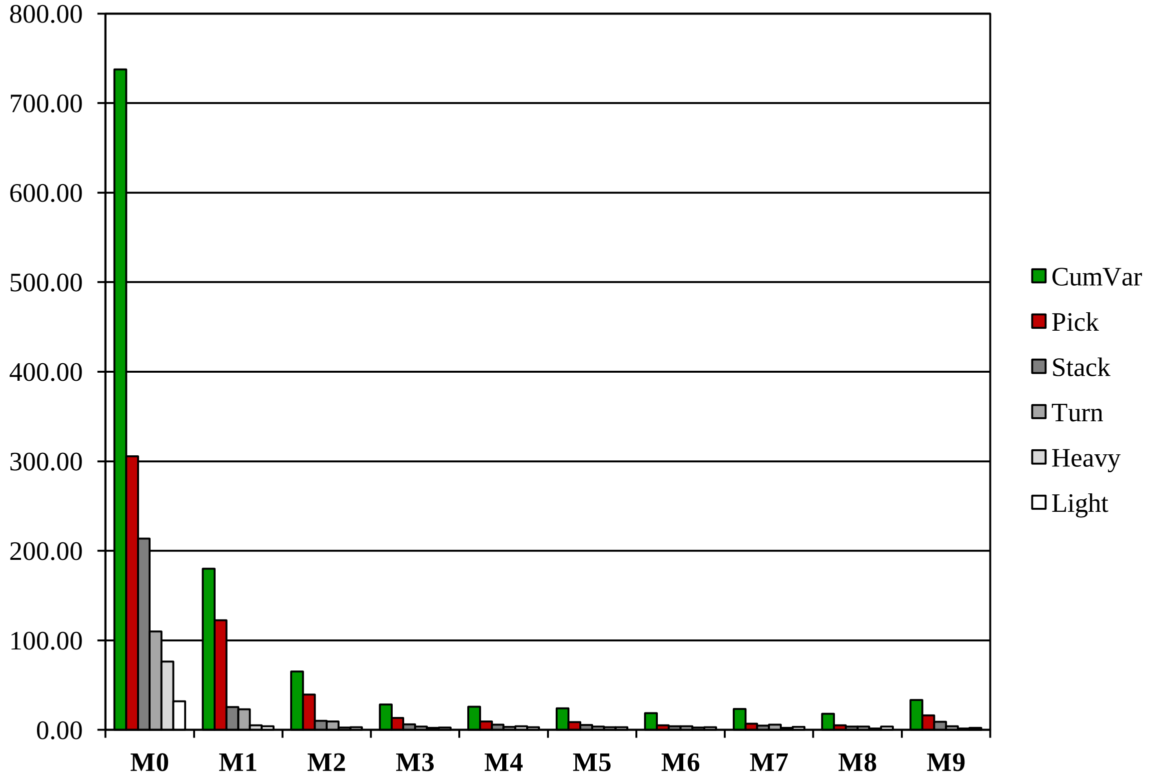

The tables below summarize the average variance of each task both between-subjects (i.e. variance per visit across patients for each task, as in Fig. S1) and within-subjects (i.e. per patient across visits for each task), respectively. The Picking Small Objects task varies greatly in both dimensions.

Average between-subjects variance

Task	*%CumVar*	*SD*
Pick (PSO)	42.38	13.88
Stack	21.61	4.48
Turn	16.55	4.59
Heavy	10.28	5.84
Light	9.18	4.21

Average within-subjects variance

Task	*Var*	*SD*
Pick (PSO)	41.64	98.66
Stack	26.56	62.85
Turn	14.95	35.24
Light	7.66	32.27
Heavy	3.35	8.37

Comparison of subtest principal component scores

As shown bellow, correlation coefficients of the first PC of PSO and each motor subtest were highly significant. Using other tasks than PSO is thus expected to give similar results than those presented in the main text.

Task	*r*	*p*
Turn PC1	0.7286	2.3544e-07
Stack PC1	0.7763	1.3231e-08
Light PC1	0.7336	1.7886e-07
Heavy PC1	0.4788	0.0016

Single-subject lesion location

Figure S1. Representative axial sections of diffusion weighted images from all patients

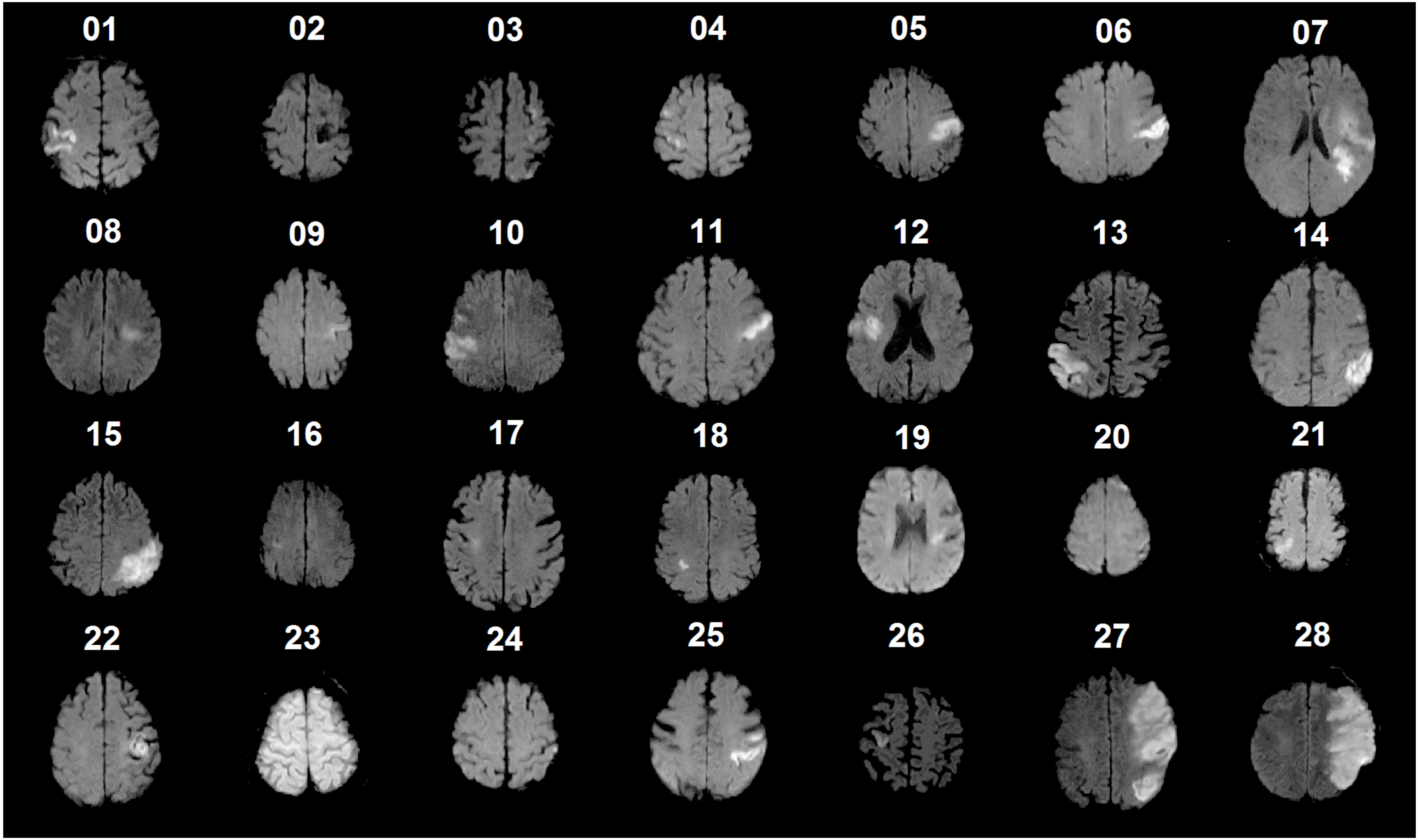

Additional structural covariance networks

Table S1 and S2 on the next pages summarize neuroanatomical regions of PC1TBM, and PC4TBM, respectively. Table S3 summarizes results of multivariate linear regression.

Table S1: Structural covariance network $PC1_{TBM}$

	Anatomical areas	Cluster size (number of voxels)	MNI coordinates (x/y/z)	Cytoarchitectonic area	Suggested functional correlate
**+ Clusters**					
1.	R Parietal operculum/SII	667	58/-20/28	OP1	Parietal network for higher order somatosensory processing
	R Inferior parietal lobule			PFop, PFt	Action observation and imitation
	R Postcentral g.			3b, 1	Somatosensory information perception (3b) and processing(1,2)
	R Heschl g.		42/-32/12	TE 1.1	No significance in the behavioral context
2.	R Inferior parietal lobule	458	40/-60/26	PGa, PGp	Language related processing
	R superior temporal g.		46/-36/6	n.a.	Spatial awareness
	R middle temporal g.		44/-52/18	n.a.	Auditory – somatosensory convergence zone
**- Clusters**					
1.	L Inferior temporal g.	481	-44/-20/-28	n.a.	Storage of upper limb movements
	L Fusiform g.		-34/-30/-26	n.a.	
2.	R Insula	459	32/-12/-2	Ig1,Ig2, Id1	Sensory feedback to motor areas
3.	L Parietal operculum/SII	134	-38/-10/14	OP3	Parietal network for higher order somatosensory processing

Table S2: Structural covariance network $PC4_{TBM}$

	Anatomical areas	Cluster size (number of voxels)	MNI coordinates (x/y/z)	Cytoarchitectonic area	Suggested functional correlate
**+ Clusters**					
1.	R Pre- and postcentral g.	806	34/-32/42	4p,3a,3b,1,2	Somatosensory guided finger movements (4p)
					SS information perception (3a,b) and processing(1,2)
	R Sup. Parietal lobule		28/-44/46	5I,7PC	Motor attention and processing (kinesthetic information
	R Intraparietal s.		34/-32/42	hIPI,hIP2, hiP3	Action related somatosensory information processing
	R Inferior parietal lobule		52/-28/30	PFop, PFcm, PFt	Action observation and imitation
		PF	Spatial and non-spatial attention		
**- Clusters**					
1.	R Precentral g.	393	52/-4/38	4a	Self-generated motor activity, secondary motor execution in response to complex sensory stimulation
	R Ventral premotor c.		34/14/34	n.a.	Space perception, action understanding and imitation
2.	R d-lat. Prefrontal c. (dorsal-posterior part)	173	42/36/22	n.a.	Action execution and working memory
3.	R. Lingual g.	150	18/-64/-12	hOC3, hOC4	Cross modal spatial attention
	R. Fusiform g.		30/-78/-12	hOC3, hOC4	
4.	R Inferior parietal lobule	140	52/-60/18	PGp	Spatial and non-spatial attention
5.	R Middle temporal g.	121	58/-50/2	n.a.	Auditory – somatosensory convergence zone

Table S3: Results of multivariate linear regression.

Full model: R^2 *= 0.692, Adj.*R^2 *= 0.604; F(6,21)= 7.87, p<.001.*

	b	*SE b*	*CI b LB*	*CI b UB*	*β*	*t*	*p*
Intercept	-32.01	14.29	-61.73	-2.29	-.16	-2.23	.04
$PC1_{TBM}$	-25.24	15.46	-57.38	6.90	-.26	-1.63	.12
$PC2_{TBM}$	49.86	23.75	0.46	99.26	.51	2.10	.05
$PC4_{TBM}$	26.45	16.30	-7.46	60.35	.26	1.62	.12
Age	0.35	0.21	-0.09	0.79	.23	1.66	.11
Lesion Volume	0.24	0.11	0.02	0.47	.45	2.23	.04
GMV Thalamus	-402.23	477.94	-1396.17	591.7	-.19	-0.84	.41

Reduced model: R^2*=0.639, Adj.*R^2*= 0.594; F(3,24)=14.18, p<1.6e-5.*

	b	*SE b*	*CI b LB*	*CI b UB*	*β*	*t*	*p*
Intercept	-9.39	3.12	-15.82	-2.95	-.27	-3.01	<.01
$PC2_{TBM}$	-26.16	19.72	-66.87	14.55	.01	-1.33	.20
Lesion Volume	0.08	0.10	-0.13	0.28	.13	0.79	.44
$PC2_{TBM}$ x Lesion Vol.	1.10	0.29	0.51	1.69	.39	3.83	<.001

Abbreviations: PC, principal component;, TBM, tensor based morphometry; GMV, gray matter volume; SE, standard error; b (β), regression coefficients (standardized); CI, 95% confidence interval; LB, lower bound; UB, upper bound

Supplemental references

[1] Mathiowetz V, Weber K, Volland G, Kashman N. Reliability and validity of grip and pinch strength evaluations. J Hand Surg Am. 1984 Mar;9(2):222-6.

[2] Jebsen RH, Taylor N, Trieschmann RB, Trotter MJ, Howard LA. An objective and standardized test of hand function. Archives of physical medicine and rehabilitation. 1969 Jun;50(6):311-9.

[3] Sunderland A, Bowers MP, Sluman SM, Wilcock DJ, Ardron ME. Impaired dexterity of the ipsilateral hand after stroke and the relationship to cognitive deficit. Stroke; a journal of cerebral circulation. 1999 May;30(5):949-55.

[4] Sunderland A. Recovery of ipsilateral dexterity after stroke. Stroke; a journal of cerebral circulation. 2000 Feb;31(2):430-3.

[5] Wetter S, Poole JL, Haaland KY. Functional implications of ipsilesional motor deficits after unilateral stroke. Archives of physical medicine and rehabilitation. 2005 Apr;86(4):776-81.

[6] Chestnut C, Haaland KY. Functional significance of ipsilesional motor deficits after unilateral stroke. Archives of physical medicine and rehabilitation. 2008 Jan;89(1):62-8.

[7] Brett M, Leff AP, Rorden C, Ashburner J. Spatial normalization of brain images with focal lesions using cost function masking. NeuroImage. 2001 Aug;14(2):486-500.

[8] Andersen SM, Rapcsak SZ, Beeson PM. Cost function masking during normalization of brains with focal lesions: still a necessity? NeuroImage. 2010 Oct 15;53(1):78-84.

[9] Ripolles P, Marco-Pallares J, de Diego-Balaguer R, Miro J, Falip M, Juncadella M, et al. Analysis of automated methods for spatial normalization of lesioned brains. NeuroImage. 2012 Apr 2;60(2):1296-306.

[10] Abela E, Missimer J, Wiest R, Federspiel A, Hess C, Sturzenegger M, et al. Lesions to primary sensory and posterior parietal cortices impair recovery from hand paresis after stroke. PLoS One. 2012;7(2):e31275.
